# Geographical patterns of malaria transmission based on serological markers for falciparum and vivax malaria in Ratanakiri, Cambodia

**DOI:** 10.1186/s12936-016-1558-1

**Published:** 2016-10-19

**Authors:** Karen Kerkhof, Vincent Sluydts, Somony Heng, Saorin Kim, Myrthe Pareyn, Laura Willen, Lydie Canier, Siv Sovannaroth, Didier Ménard, Tho Sochantha, Marc Coosemans, Lies Durnez

**Affiliations:** 1 Department of Biomedical Sciences, Institute of Tropical Medicine, Antwerp, Belgium; 2 Department of Biomedical Sciences, University of Antwerp, Antwerp, Belgium; 3 Department of Biology, University of Antwerp, Antwerp, Belgium; 4 National Centre for Parasitology, Entomology and Malaria Control, Phnom Penh, Cambodia; 5 Molecular Epidemiology Unit, Institut Pasteur du Cambodge, Phnom Penh, Cambodia

**Keywords:** Malaria, Serological markers, Geographical patterns, Malaria pockets, Heterogeneous transmission, Cambodia

## Abstract

**Background:**

Malaria transmission is highly heterogeneous, especially in low endemic countries, such as Cambodia. This results in geographical clusters of residual transmission in the dry, low transmission season, which can fuel the transmission to wider areas or populations during the wet season. A better understanding of spatial clustering of malaria can lead to a more efficient, targeted strategy to reduce malaria transmission. This study aims to evaluate the potential of the use of serological markers to define spatial patterns in malaria exposure.

**Methods:**

Blood samples collected in a community-based randomized trial performed in 98 high endemic communities in Ratanakiri province, north-eastern Cambodia, were screened with a multiplex serological assay for five serological markers (three *Plasmodium falciparum* and two *Plasmodium vivax)*. The antibody half-lives range from approximately six months until more than two years. Geographical heterogeneity in malaria transmission was examined using a spatial scan statistic on serology, PCR prevalence and malaria incidence rate data. Furthermore, to identify behavioural patterns or intrinsic factors associated with malaria exposure (antibody levels), risk factor analyses were performed by using multivariable random effect logistic regression models. The serological outcomes were then compared to PCR prevalence and malaria incidence data.

**Results:**

A total of 6502 samples from two surveys were screened in an area where the average parasite prevalence estimated by PCR among the selected villages is 3.4 %. High-risk malaria pockets were observed adjacent to the ‘Tonle San River’ and neighbouring Vietnam for all three sets of data (serology, PCR prevalence and malaria incidence rates). The main risk factors for all *P. falciparum* antigens and *P. vivax* MSP1.19 are age, ethnicity and staying overnight at the plot hut.

**Conclusion:**

It is possible to identify similar malaria pockets of higher malaria transmission together with the potential risk factors by using serology instead of PCR prevalence or malaria incidence data. In north-eastern Cambodia, the serological markers show that malaria transmission occurs mainly in adults staying overnight in plot huts in the field. Pf.GLURP.R2 showed a shrinking pocket of malaria transmission over time, and Pf.MSP1.19, CSP, PvAMA1 were also informative for current infection to a lesser extent. Therefore, serology could contribute in future research. However, further in-depth research in selecting the best combination of antigens is required.

**Electronic supplementary material:**

The online version of this article (doi:10.1186/s12936-016-1558-1) contains supplementary material, which is available to authorized users.

## Background

The annual malaria incidence and mortality have steadily declined in the Greater Mekong Subregion over the past 15 years [1]. In particular, in the Kingdom of Cambodia malaria cases were reduced by more than 75 %, due to the improvements in malaria control by the National Malaria Control Programme, such as free distribution of long-lasting insecticidal nets (LLINs), performing better case management (i.e. diagnosis by RDT and treatment with ACT) [2, 3]. Although malaria transmission in Cambodia nowadays is low [4, 5], still 21 out of 25 provinces are endemic, of which the northeast region (Ratanakiri) accounts for more than 70 % of the malaria burden [3, 6]. Moreover, a shift towards more heterogeneous malaria transmission has been observed. This results in areas that support malaria transmission, which are referred to as foci [7, 8]. Within these foci, elevations in malaria transmission in small areas (sometimes <1 km^2^ [7]) or populations can be identified, which are respectively called hotspots or hotpops, presenting a higher risk of infection as compared to the rest of the focus [7, 9]. Some studies have shown that stable hotspots with a permanent transmission of the parasite over consecutive dry seasons mainly consist of asymptomatic carriers [10]. Consequently, in low endemic areas as Cambodia, in the wet high transmission season, when the malaria vector population expands, these remaining reservoirs tend to fuel malaria transmission to surrounding areas or populations [7, 11]. Despite considerable malaria control efforts in Cambodia, this persisting transmission makes it impossible to eliminate malaria by current control strategies. Therefore, it is necessary to move forward from these strategies by creating a novel strategic plan based on the understanding of the hotspots/hotpops [3, 12]. It is assumed that there will be no persistence of malaria transmission once these recurrent sources of infection are eliminated by targeted interventions to these hotspots and hotpops [3, 8].

So far, hotspot identification has been carried out by various approaches, including microscopy, rapid diagnostic tests (RDTs), PCR detection and entomological surveys. Microscopy and RDTs cannot detect low-density infections [13]. In addition, among the PCR-based assays, real-time PCR is a highly sensitive technique capable of detecting higher numbers of infected individuals, including asymptomatic infections. However, this technique has a couple of drawbacks when used in the field e.g. high costs and complexity of its applicability [13]. Serological markers of malaria exposure, specifically antibodies (Abs) against *Plasmodium* antigens (Ags), are appropriate to use when detecting stable hotspots of malaria transmission in low endemic areas [7]. These Ab-responses increase by cumulative exposure and the longevity of the Abs depends on the Ag [7]. Therefore, this method can provide an indication of past and recent malaria exposure that can be used to pick up temporal and spatial trends in malaria transmission [7, 14]. Moreover, previous elimination programs have already observed that the absence of Ab-titers in the youngest age-groups could be used as proof of the cessation of malaria transmission [14]. However, in Southeast Asia, primo infection by malaria parasites may be delayed to adolescence, due to behavioural and occupational activities [15]. Serology has already been used to detect spatial trends by previous studies in high endemic settings [8, 11, 16–20]. On the contrary, the serological value for detecting spatial clustering of malaria exposure in low endemic areas has not yet been completely confirmed [7, 8].

The proposed study aims to further validate five *Plasmodium* markers for their potential to detect recent infection [21] by defining spatial patterns in malaria exposure over two different surveys in comparison with PCR prevalence and malaria incidence data. As in this study, community (= cluster) based data were used, the outcomes were defined as ‘malaria pockets’ [22] referring to an area in between a hotspot (<1 km^2^ [7]) and a foci (>1 province) where the malaria exposure is higher than the surrounding areas.

## Methods

### Study area

Ratanakiri province (13°44′N, 107°0′E) bordering Lao PDR and Vietnam, is located 520 km from Phnom Penh in the northeast of Cambodia. The area has a monsoonal climate, resulting in perennial malaria transmission with a peak during the rainy season (April until October) [5, 15]. Ratanakiri has a population of 149,997 individuals spread over 240 villages [23], of which approximately 70 % is living in the highlands and 30 % in the urbanized towns. This study area is largely inhabited by the ethnic minority (e.g. Jarai, Kreung, Tumpuon) as opposed to the Khmer in the rest of the Country. The ethnic minority generate revenue by subsistence slash-and-burn farming. Therefore, they own plot huts located near or inside the forests, where they stay during most of the rainy season [23, 24].

### Sample collection

Samples used were derived from a community (=cluster) based randomized trial (MalaResT project—NCT01663831) that aims to evaluate the effectiveness of topical repellents, in addition to long-lasting insecticidal nets, on malaria prevalence and incidence [5, 21, 25]). For the purpose of this study two cross-sectional surveys carried out in November 2012 and 2013 were included. No differences were observed between the control and the intervention arm for PCR prevalence, serological indicators and malaria incidence. Blood samples were collected by a finger prick on filter paper, and immediately screened by real-time PCR to determine the presence or absence of parasites [5]. In the MalaResT project 65 people per community were randomly selected, and in case of a low attendance rate an additional set of 15 randomly selected people was added to reach at least 50 participants per community. Throughout the entire sampling process an over 70 % success rate was reached, in which all age groups were proportionally covered [23]. From a total population of 48,838 individuals residing in all 113 villages grouped in 98 communities (i.e. clusters; 88 single villages +25 neighbouring villages having a distance of <1 km were grouped into 10 communities), a total of 6640 and 6715 were randomly recruited by community in November 2012 and 2013 of which respectively 4996 and 5431 were sampled [23]. Serology was performed on 3264 (2012) and 3238 (2013) on randomly selected samples. The PCR *Plasmodium* prevalence was 4.9 and 3.4 % in November 2012 and 2013 respectively (Table 1) [23].

**Table 1:** Descriptive statistics of the study site and the inhabitants

Characteristics	November 2012	November 2013
No. samples	3264	3238
Age		
2–5	468 (14.3 %)	423 (13.1 %)
6–15	998 (30.6 %	968 (29.9 %)
16–50	1793 (54.9 %)	1847 (57.0 %)
Median	18	19
Gender		
Male	1610 (49.9 %)	1616 (49.9 %)
Female	1653 (50.6 %)	1622 (50.1 %)
PCR prevalence		
All 98 communities	4.86 %	3.41 %
Pf	2.22 %	1.20 %
Pv	2.94 %	2.24 %
Mean MFI-values Pf markers		
CSP	208	878
Pf.GLURP.R2	3064	2075
Pf.MSP1.19	259	289
Mean MFI-values Pv markers		
Pv.MSP1.19	277	314
Pv.AMA1	822	845

### Serology

Initially, each Ag was coupled to paramagnetic beads (MagPlex microspheres, Luminex Corp, Austin, TX, USA) as described earlier [26]. All beads with different Ags were put together to prepare a microsphere working mixture at a concentration of 1000 beads/Ag/well. Bloodspot samples were then analysed in 96-well plates in duplicate. Positive controls (pool of four *P. falciparum* and two *P. vivax* infected individuals), negative control serum and blanco (PBS-CR) were added in duplicate to each 96-well plate [21]. The MAGPIX®-system was set for reading a minimum of 400 beads per spectral address and results were expressed as median fluorescent intensity (MFI) [21].

### Antigen selection

The selection of the five Ags used in this study was based on the half-lives estimated on 20 different Ags previously [26]. For *P. falciparum* three out of the six serological markers that were most likely to be reflective for recent exposure were chosen. These were Pf.GLURP.R2, Pf.MSP1.19 and CSP, showing a half-life of respectively six months, ~8 months and ~1 year. For *P. vivax,* the shortest Ab half-life found was more than 1.5 years (PvAMA1 and Pv.MSP1.19).

### Malaria incidence

Passive case detection (confirmed malaria cases) was reinforced for the purpose of the study and rely on the National Health System (Village Malaria Workers, health centres and hospitals) [6]. For each case the living place was recorded in the database. Incidence was estimated by community.

### Statistical analysis

The serology outcome data (median fluorescent intensity—MFI) were processed and analysed in R-version 3.1.0 [27]. Spatial clustering of serological data was detected for each survey by using spatial scan statistic (SaTScan) [28, 29], which is the preferred software in low risk settings according to Aamodt et al. [25, 30]. The spatial scan statistic was used on the natural logarithm transformed median fluorescent intensity values (ln(MFI)—normal probability model) to define spatial patterns in malaria exposure and to compare these outcomes to malaria pockets obtained by the PCR prevalence (binominal, Bernoulli model) and malaria incidence data recorded by community in all ages (counts, Poisson model).

The R package *‘rsatscan’* [31] was used to prepare the data, and R package *‘PlotKML’* [32] was used to be able to plot the 98 communities on the Google Earth images created with SaTScan. By using the spatial scan statistic, SaTScan version 9.4.2, 64-bit [28, 29], the study area (province of Ratanakiri) was systematically screened for circular windows of higher MFI values. Ratanakiri is a large area (10,782 km^2^). To avoid detection of too large pockets that cover almost the entire area and hide small homogeneous malaria pockets within the larger pockets, the maximum of the total population at risk within a community was set at 20 %, in accordance with the observations in the study of Mosha et al. [9]. This allows that both small and large malaria pockets can be detected [33]. Observed and expected means of ln (MFI) values inside and outside each window at each location were calculated [8, 11, 29]. The areas with the maximum likelihood were defined as the malaria pockets [11]. These pockets were then examined based on 999 Monte Carlo simulations. The malaria pockets were considered statistically significant with a *p* value <0.05. Circular windows were visualized separately for November 2012 and 2013. The median age inside and outside the malaria pockets was estimated for the serological data.

The outcomes perceived with the serological markers were compared to PCR prevalence (performed on the same surveys) and malaria incidence rate (recorded the same year) [23]. When these outcomes correspond to the results observed in this study, ongoing malaria transmission at the localities could be confirmed. Therefore, these malaria pockets were detected by using a similar approach as detailed above, but with the PCR prevalence data (Bernoulli model) from November 2012 and 2013, whereby the maximum allowed population size was set to 20 % as well. Thereby, pockets were significant with a p-value below 0.05. The same was done for the malaria incidence data of symptomatic cases based on the health information system (Poisson model) from 2012 and 2013. Pockets were considered significant when presenting a p-value below 0.05. A sensitivity and specificity analysis was done to assess the prediction of serological based malaria pockets in comparison to PCR or Incidence malaria pockets.

Moreover, a risk factor analysis was performed to detect a pattern in behavioral or intrinsic factors associated with high endemic pockets. The risk factor analysis was carried out in various steps as described previously [25], with minor adjustments. Firstly, a univariate analysis was performed on all explanatory variables: age, gender, ethnicity (khmer vs ethnic minority), axillary temperature, plot hut (a human behaviour associated with indigenous farming), sleeping in the forest (indoor/outdoor) and repellent use [control (only bed net) *vs* intervention (bed net and repellent use)] [25]. The risk factors were analysed by fitting linear mixed effect models (lmer function in the *‘lme4’* package applied in R version 3.1.0 [34]) with ln(MFI) values as outcome variable taking into account community (=cluster) within survey (year) as random effect [35]. Incidence rate ratios (IRR) and 95 % confidence intervals (95 % CI) were estimated by exponentiation of the model coefficients per variable and per Ag. Statistical significance was evaluated based on the p-values below 0.10. Next, residual plots were made to evaluate how well the models fitted the data and how the data meet the assumptions of the model. To check whether variables needed to be omitted due to co-linearity for the multivariable model, the Variance inflations factors (VIF) of each linear model were evaluated (R packages *‘MASS’* and *‘car’* [36, 37]). Lastly, multivariable random effect logistic regression models were fitted (lmer function in R package *‘lme4’* [34]). Model selection through stepwise backward deletion, starting from the full model, was built based on the outcomes of the univariate analysis to define independent relations between the variables and the strength of the Ab-response [38, 39].

## Results

For this study a random selection of 6502 samples were screened with the Luminex technology after being tested for parasite infection using real-time PCR. An overview of the descriptive statistics is given in Table 1.

### Geographical clustering based on serology (*Plasmodium falciparum* and *Plasmodium vivax* antigens)

**Fig. 1:**
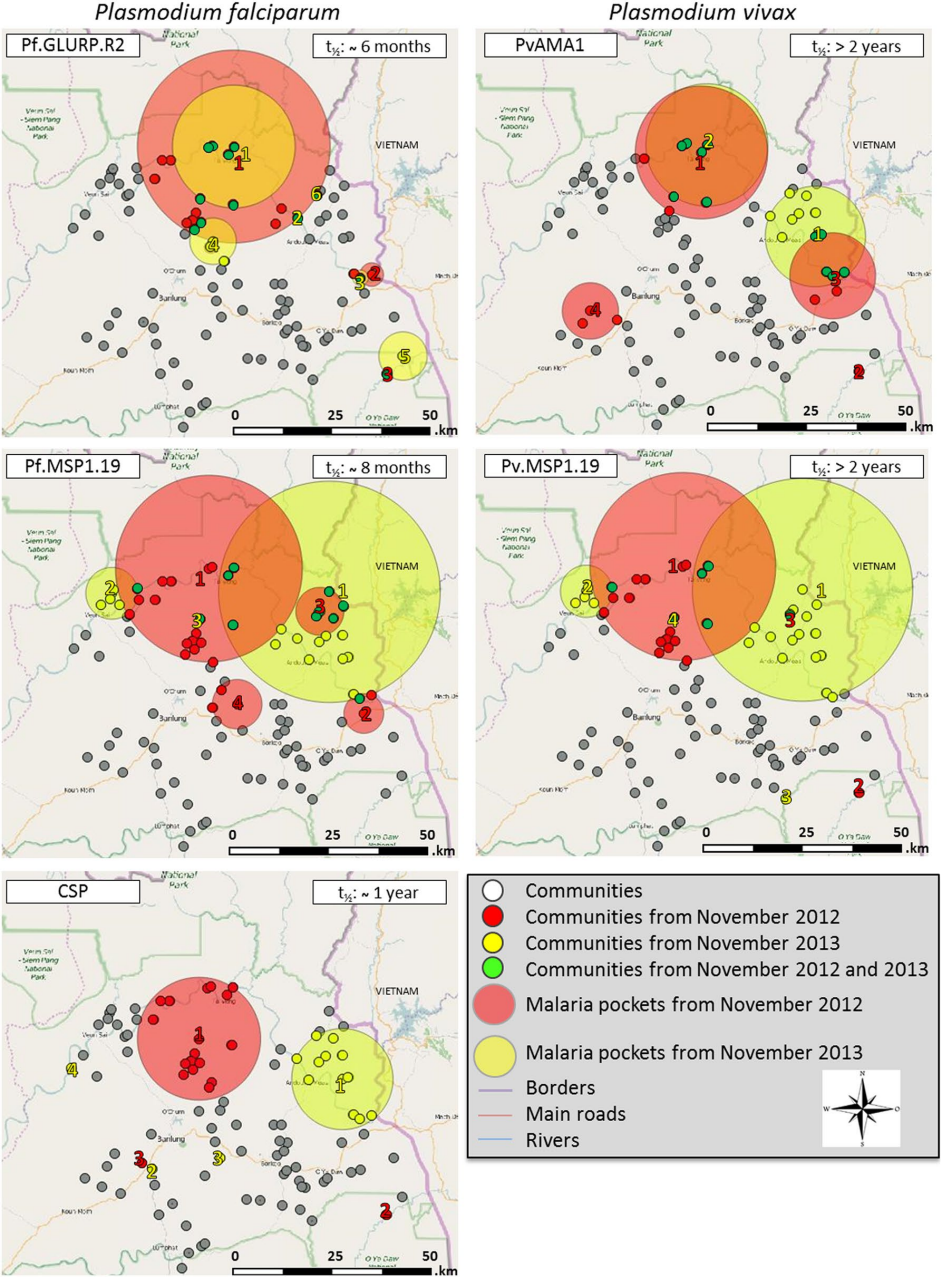
Malaria pockets with higher ln-MFI values for antibodies against *Plasmodium falciparum* and *Plasmodium vivax* in Ratanakiri, Cambodia. *White dots* indicate all 98 communities that were included in this study. *Red dots* (November 2012), *yellow squares* (November 2013) and the *green dots* (November 2012 and 2013) specify the communities within the most likely malaria pockets. The *large red* (November 2012) and *yellow* (November 2013) *circles* are the statistically significant malaria pockets in which higher *Plasmodium* antibody intensity was detected by spatial scan statistics (p < 0.005)

A first pocket was detected around the most northerly point of the ‘Tonle San River’ for all five Ags (*P. falciparum* and *P. vivax*) in November 2012 (Fig. 1; Table 2). For four out of five Ags (CSP, Pf.MSP1.19, Pf.GLURP.R2 and Pv.MSP1.19), the mean geographical radius was estimated 21.33 km, consisting of approximately 18 communities (p = 0.001). Moreover, for PvAMA1 one pocket was located at the same place, but showed a smaller radius of 16.45 km, with 8 communities (p = 0.001). In the same year, another pocket (Fig. 1; Table 2) was perceived around the ‘Tonle San River’ nearby the border with Vietnam for Pf.MSP1.19, Pf.GLURP.R2 and PvAMA1, with a radius of respectively 5.97 km (5 communities), 2.90 km (2 communities) and 10.67 km (7 communities) (p = 0.001). For the remaining two Ags (CSP and PvMSP1.19), a malaria pocket was detected adjacent to Vietnam as well, but was distant from the ‘Tonle San River’. These malaria pockets had a radius of 0 km, as these consist of only one community (p = 0.002). PvAMA1 showed a fourth pocket more inland (3 communities, radius 7 km), and for Pf.MSP1.19 a pocket close to the capital of Ratanakiri, ‘Ban Lung’, was observed (1 community, radius 0 km).

**Table 2:** Spatial malaria pockets with higher level of antibodies against *Plasmodium falciparum* and *Plasmodium vivax* antigens detected by SaTScan v9.4.2 in Ratanakiri, Cambodia

Antigens	Malaria pockets	Communities (out of 98)	Year	Total # persons tested per Ag	# of persons per pocket	% of people living in pocket	Radius (km)	Mean ln-MFI inside pocket	Mean ln-MFI outside pocket	Median age inside pocket	Median age outside pocket	Log likely-hood ratio	p-value
CSP	3	17	Nov 2012	3067	540	17.6	15.15	5.69	5.37	20	18	12.88	0.001
		1			18	0.6	0	6.84	5.42	16	18	10.54	0.002
		1			23	0.7	0^a^	6.53	5.43	20	18	8.15	0.018
	4	12	Nov 2013	2880	335	11.6	12.46	5.77	5.41	16.5	18	11.67	0.001
		1			11	0.4	0^a^	6.95	5.45	25	18	7.81	0.018
		1			20	0.7	0^a^	6.54	5.45	16	18	7.48	0.026
		1			5	0.2	0^a^	7.59	5.45	20	20	7.20	0.034
Pf.MSP1.19	4	19	Nov 2012	2921	563	19.3	23.18	5.46	5.21	20	18	91.87	0.001
		3			88	3.0	4.95	5.64	5.28	22	18	11.59	0.001
		5			163	5.6	5.97	5.53	5.28	15	18	10.05	0.001
		3			84	2.9	6.16	5.58	5.29	18	18	7.68	0.018
	4	19	Nov 2013	2757	549	19.9	27.49	5.68	5.31	16	19	60.45	0.001
		6			162	5.9	6.44	5.69	5.37	18	18	16.10	0.001
		1			26	0.9	0^a^	6.05	5.38	26	18	11.23	0.001
		1			32	1.2	0^a^	5.88	5.38	23	18	7.83	0.017
Pf.GLURP.R2	4	18	Nov 2012	3142	601	19.1	23.79	7.18	6.60	19	18	26.52	0.001
		2			59	1.9	2.90	8.01	6.69	20	18	16.77	0.001
		1			26	0.8	0^a^	8.35	6.70	16	18	11.49	0.002
		1			37	1.2	0^a^	7.89	6.70	14	18	8.50	0.009
	6	6	Nov 2013	2953	177	6.0	15.15	7.32	6.54	19	18	19.29	0.001
		1			40	1.4	0^a^	7.87	6.57	19	18	12.90	0.002
		2			44	1.5	1.92	7.60	6.57	20	18	8.87	0.010
		5			166	5.6	5.74	7.07	6.56	19	18	7.93	0.020
		2			68	2.3	5.97	7.33	6.57	18	18	7.33	0.032
		1			20	0.7	0^a^	7.97	6.57	13	18	7.33	0.032
PvAMA1	4	8	Nov 2012	3030	264	8.7	16.45	6.64	6.20	20	18	32.10	0.001
		1			21	0.7	0^a^	7.61	6.23	16	18	27.25	0.001
		7			200	6.6	10.67	6.52	6.21	18.5	18	11.74	0.001
		3			56	1.8	7.00	6.70	6.23	20	18	8.35	0.025
	2	12	Nov 2013	2801	324	11.6	12.46	6.66	6.25	16.5	18	36.73	0.001
		6			182	6.5	15.15	6.64	6.28	19	18	16.39	0.001
Pv.MSP1.19	3	19	Nov 2012	2915	564	19.3	23.18	5.64	5.24	20	18	74.18	0.001
		1			12	0.4	0^a^	6.41	5.31	16	18	14.45	0.001
		1			30	1.0	0^a^	5.99	5.31	13	18	13.95	0.001
	4	19	Nov 2013	2762	547	19.8	27.49	5.69	5.35	16	19	48.55	0.001
		6			161	5.8	6.44	5.71	5.40	18	18	13.32	0.001
		1			34	1.2	0^a^	5.94	5.41	23	18	8.54	0.012
		1			27	0.97	0^a^	5.96	5.41	26	18	7.30	0.029

The MFI values inside and outside the pockets are based on the natural logarithm

^a ^A single village was selected as an area with a higher risk to *Plasmodium* infection, and therefore showing a radius of 0 km

In November 2014, a malaria pocket was observed again for both Ags Pf.GLURP.R2 and Pv.AMA1, located at the northerly point of the ‘Tonle San River’ (Fig. 1; Table 2). For Pf.GLURP.R2, the pocket size was reduced by approximately 35 % (from 18 to 6 communities in the malaria pockets) as compared to November 2012, whereas for PvAMA1 the radius was reduced by 8 % (from 8 to 6 communities in the malaria pockets), however, for PvAMA1 the communities neighbouring Vietnam increased with 16.8 % (from 7 to 12 communities in the malaria pockets) and showed a more northern spread compared to November 2012. A fourth pocket for Pf.GLURP.R2 was perceived closer to ‘Ban Lung’ (5 communities, radius 7.54 km).

A different pattern was perceived for CSP, showing an eastward shift (37.35 km) in the malaria transmission locality between November 2012 and 2013 (Fig. 1; Table 2). This resulted in only one large malaria pocket (>1 community) in November 2013, neighbouring Vietnam and having a radius of 12.46 km consisting of 12 communities (p = 0.001).

Primary malaria pockets obtained for Pf.MSP1.19 and Pv.MSP1.19 were similar in November 2012 and 2013 (Fig. 1; Table 2) having a mean radius of 23.18 km, and 19 communities (p < 0.001) with a shift of the main pocket towards the Vietnamese border in November 2013 (mean radius of 27.49 km, 19 communities (p < 0.001). Additionally, in November 2013 a smaller malaria pocket appeared for both Ags with a mean radius of 6.44 km, including 6 communities (p < 0.001) more to the northwest side of the ‘Tonle San River’.

For all Ags the age distribution inside and outside the pockets was shown to be similar (Table 2).

### Confirmation of the ongoing malaria transmission

**Fig. 2:**
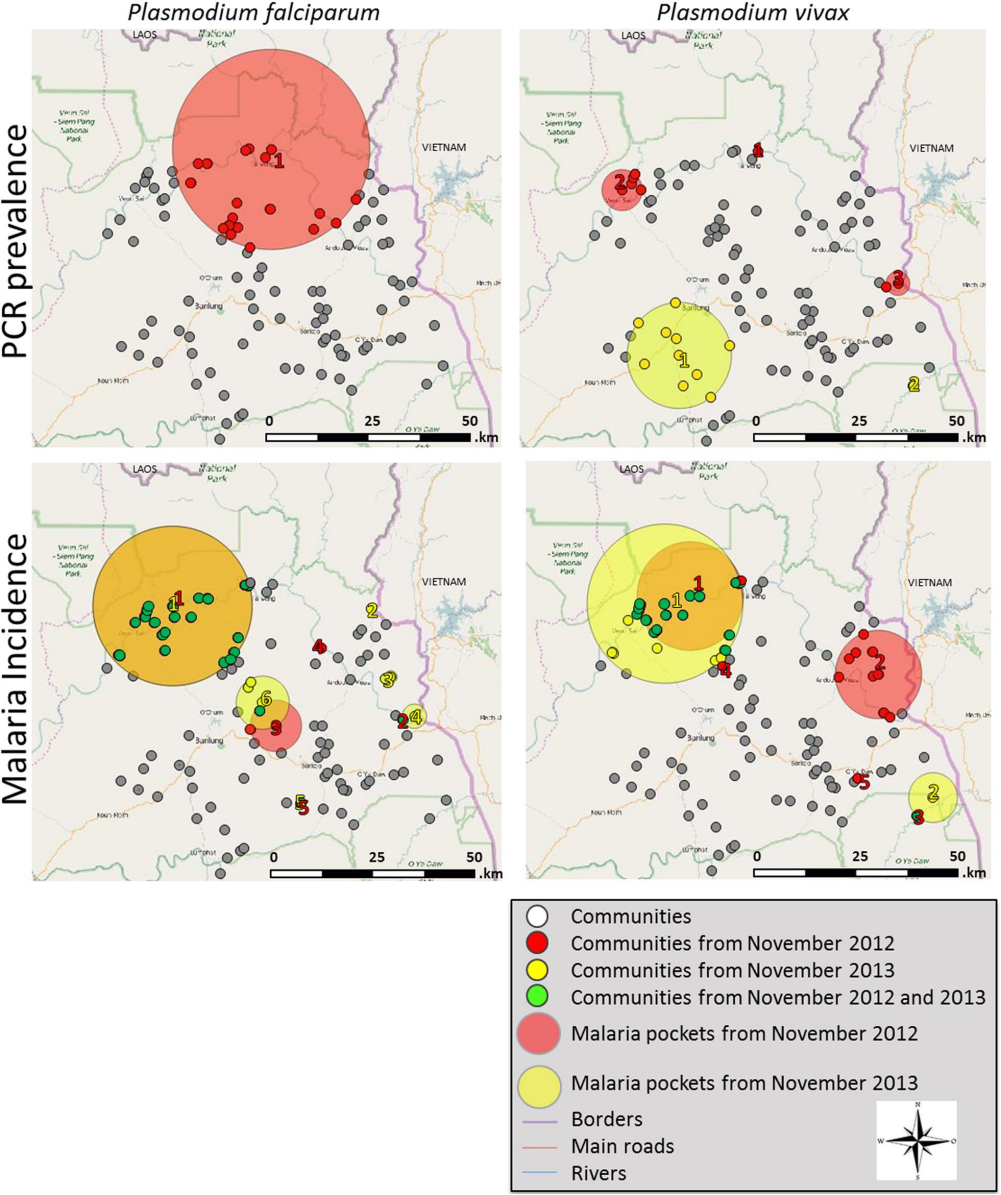
Malaria pockets with higher PCR-prevalence and Incidence rate of *Plasmodium falciparum* and *Plasmodium vivax* mapped in Ratanakiri, Cambodia. *White dots* indicate all 98 communities that were included in this study. *Red dots* (November 2012), *yellow squares* (November 2013) and the *green dots* (November 2012 and 2013) specify the villages within the most likely malaria pockets. The *large red* (November 2012)) and *yellow* (November 2013) *circles* are the statistically significant malaria pockets in which the higher *Plasmodium* PCR prevalence or incidence rates were detected by spatial scan statistics (p < 0.0125)

*Plasmodium* prevalence and malaria incidence were determined by PCR (prevalence per survey, Fig. 2; Table 3) [25] and passive case detection (yearly incidence rate, Fig. 2; Table 4). The same pockets as the ones detected by serological markers were observed with PCR in the most northerly area of the ‘Tonle San River’ in November 2012 for *P. falciparum*, whereas no PCR prevalence pockets were detected in November 2013. Looking at the *P. falciparum* malaria incidence data, malaria pockets did not move between 2012 and 2013, but slightly shifted to the western part of the river as compared to the main pocket acquired with PCR prevalence and serological markers.

**Table 3:** Spatial malaria pockets with higher PCR prevalence of *Plasmodium falciparum* and *Plasmodium vivax* species detected by SaTScan v9.4.2 in Ratanakiri, Cambodia

*Plasmodium* species	Malaria pockets	Communities (out of 98)	Year	Radius (km)	Population	Observed cases	Expected cases	Relative risk	Log likelihood ratio	p-value
*P. falciparum*	2	19	Nov 2012	23.92	953	46	21.36	2.96	14.75	<0.05
		1		0^a^	53	7	1.19	6.22	7.10	<0.05
	0		Nov 2013							>0.05
*P. vivax*	3	1	Nov 2012	0^a^	52	12	1.55	8.33	15.66	<0.05
		5		4.91	259	22	7.72	3.17	9.93	<0.05
		2		2.90	103	13	3.07	12.6	9.69	<0.05
	2	1	Nov 2013	0^a^	61	10	1.38	7.79	12.15	<0.05
		7		12.69	538	29	12.18	2.81	9.98	<0.05

^a^A single village was selected as an area with a higher risk to *Plasmodium* infection, and therefore showing a radius of 0 km

**Table 4:** Spatial malaria pockets with higher Incidence of Vivax and Falciparum malaria detected by SaTScan v9.4.2 in Ratanakiri, Cambodia

*Plasmodium* species	Malaria pockets	Communities (out of 98)	Year	Radius (km)	Population	Observed cases	Expected cases	Relative risk	Log likelihood ratio	p-value
*P. falciparum*	5	27	2012	19.08	9594	1113	527.11	2.92	335.44	<0.05
		1		0^a^	244	57	13.41	4.32	39.27	<0.05
		3		6.16	699	102	38.40	2.72	36.82	<0.05
		1		0^a^	288	44	15.82	2.81	16.97	<0.05
		1		0^a^	223	33	12.25	2.71	12.03	<0.05
	6	27	2013	19.08	9594	580	257.69	3.26	204.42	<0.05
		1		0^a^	450	47	12.09	4.00	29.39	<0.05
		2		1.50	535	41	14.37	2.91	16.63	<0.05
		2		2.90	876	55	23.53	2.40	15.62	<0.05
		1		0^a^	223	22	5.99	3.72	12.71	<0.05
		5		6.36	325	21	8.73	2.43	6.22	<0.05
*P. vivax*	5	19	2012	13.01	6752	464	201.37	2.92	154.77	<0.05
		9		10.59	3408	173	101.64	1.80	22.59	<0.05
		1		0^a^	283	30	8.44	3.61	16.64	<0.05
		1		0^a^	222	24	6.62	3.67	13.63	<0.05
		1		0^a^	368	26	10.97	2.39	7.48	<0.05
	2	27	2013	19.08	9594	366	154.30	3.60	145.45	<0.05
		2		5.97	386	31	6.21	5.16	25.46	<0.05

^a^A single village was selected as an area with a higher risk to *Plasmodium* infection, and therefore showing a radius of 0 km

For *P. vivax* sero-reactive pockets were located nearby the ‘Tonle San River’ and the border of Vietnam in November 2012. Malaria pockets detected by PCR prevalence data show a shift between the surveys from the river-side towards the south (inland) of Ratanakiri in November 2013, the same pocket was observed for PvAMA1 in November 2012. This shift to the south is also seen with the malaria incidence data but on a different locality namely at the border of Vietnam. However, in 2013 (malaria incidence data) the main pocket persisted at the northern site of the ‘Tonle San River’, which was not the case for PCR prevalence data.

Overall, malaria pockets found by analysing malaria incidence data and ln(MFI) data are similar. The largest serologically measured pocket of each Ag consistently overlaps with the largest incidence based pockets. The malaria pockets adjacent to Vietnam overlap for all three approaches. Moreover, serologically obtained pockets from November 2012 show overlap with both *P. falciparum* and *P. vivax* pockets found by PCR. In November 2013, no significant PCR prevalence based pockets were observed for *P. falciparum* and the largest PCR prevalence based pocket for *P. vivax* was located more inland.

### Sensitivity and specificity analysis (Additional file 1: Table S1)

Serology with the three *P. falciparum* Ags provides a relative good specificity (>74 %) and sensibility (>72 %) to identify positive PCR falciparum pockets. This was particularly true in November 2012 with the Pf.GLURP.R2 Ag having 95 % for both specificity and sensitivity. As no PCR falciparum pockets could be identified for November 2014, sensitivity could not be calculated. To predict *P. falciparum* incidence pockets, serological data provide a much lower sensitivity (between 13 and 60 %) although specificity was around 80 %. Serological data with the two *P. vivax* Ags provides a low sensitivity (between 0 and 38 %) but a relatively high specificity (69 and 82 %) to predict PCR vivax pockets. Results were slightly better in predicting *P. vivax* incidence pockets (sensitivity 10 and 57 %; specificity around 80 %). For comparison *P. falciparum* and *P. vivax* incidence data to predict PCR falciparum and vivax pockets sensitivity was around 50 % in 2012, but only 10 % in 2013 for *P. vivax*. Specificity lies between 70 and 80 %.

### Risk factor analysis

Significant variables (age, gender, ethnicity, plot hut and sleeping in the forest) observed in the univariate analysis (Additional file 2: Table S2) were further explored in the multivariable regression model (Table 5). In the full model used for the multivariable analysis age, gender, ethnicity, plot hut and sleeping in the forest were included as dependent variables, and community within survey as random effect. Axillary temperature and repellent were not significant for all five Ags in the univariate exploration and were, therefore, not included.

**Table 5:** Multivariable analysis of the selected risk factors associated with the seroprevalence after the univariate analysis and AIC model selection procedure

	*Plasmodium falciparum*			*Plasmodium vivax*	
Malariometric variable	CSP	Pf.MSP1.19	Pf.GLURP.R2	PvAMA1	Pv.MSP1.19
Variable level	IRR [LCI–UCI]	IRR [LCI–UCI]	IRR [LCI–UCI]	IRR [LCI–UCI]	IRR [LCI–UCI]
Age (years)					
2–5	Reference	Reference	Reference	Reference	Reference
6–15	1.44 [1.33–1.57]	1.30 [1.23–1.38]	2.83 [2.54–3.15]	1.40 [1.31–1.50]	1.21 [1.14–1.28]
16–50	5.44 [5.03–5.89]	1.68 [1.59–1.78]	13.33 [12.02–14.77]	1.98 [1.86–2.16]	1.50 [1.42–1.59]
>50	11.82 [10.61–13.15]	2.01 [1.86–2.16]	17.90 [15.55–20.61]	2.79 [2.55–3.05]	1.99 [1.84–2.15]
Gender					
Male	Reference	Reference	–	–	Reference
Female	1.06 [1.01–1.12]	1.04 [1.00–1.08]	–	–	1.06 [1.02–1.10]
Ethnicity					
Khmer	Reference	Reference	Reference	Reference	Reference
Ethnic Minority (EM)	2.72 [2.38–3.12]	1.33 [1.21–1.47]	3.70 [3.11–4.41]	1.31 [1.17–1.47]	1.18 [1.07–1.31]
Overnight plothut					
No	Reference	Reference	Reference	–	Reference
Yes	1.12 [1.06–1.19]	1.07 [1.03–1.11]	1.24 [1.15–1.33]	–	1.04 [1.00–1.09]
Overnight forest					
No	Reference	–	–	–	Reference
Yes	1.14 [1.05–1.23]	–	–	–	1.07 [1.01–1.14]

^a^ *IRR* Incidence rate ratio that indicates for how much (if > 1) or less (if < 1) the risk factors affect the data obtained in survey 2 (2012) and survey 4 (2013). This is performed in respect to the reference category and LCI and UCI representing the lower and upper 95 % confidence intervals based on the total sample size of n = 6 502 individuals from 98 communities. p-value <0.10. Missing values were not significant

Differences in the presence of Abs were perceived between age categories for all Ags. Ethnic minority showed a higher presence of Ab-levels for all Ags (mean IRR 2.051 95 % CI [1.788–2.354]). Significant differences were observed for ‘overnight stay in a plot hut’ (p < 0.10) with a mean IRR and 95 % CI of 1.126 [1.067–1.188]. Furthermore, gender appeared to be significantly different for CSP, Pf.MSP1.19 and Pv.MSP1.19, and sleeping in the forest for CSP and Pv.MSP1.19. However, the IRR of these last two risk factors are so close to one, that this is probably negligible.

## Discussion

Methods that can identify stable areas of transmission over time are suggested to be most effective for assessing geographical variations in malaria exposure. Therefore, Ab-responses acquired with cumulative malaria exposure, measured over several seasons, were recommended for implementation in geographical clustering analyses [7]. During the ‘90s geographical cluster analyses mainly relied on symptomatic cases with accurate details about the place of infection and/or residence [20, 40], and were merely based on passive case detection (PCD) [25]. It was not until the 21th century that due to a lack of information about the parasite reservoir in asymptomatic cases [10], new studies arose focussing on the spatial distribution estimated with PCR-prevalence data of species-specific geographical areas of infections based on asymptomatic carriers [25]. This approach was especially important in countries with a low endemicity where the majority of infected people are asymptomatic carriers [20]. Another innovative approach is the application of serological markers in defining these geographical areas. Serology already proved its ability to improve predictions of low transmission risk [26, 41].

Where most studies only focused on PfAMA1 and PfMSP1.19 [8, 11, 16–19], the advantage of this study is the amount of additional Ags from both falciparum and vivax malaria investigated compared to most other studies. Only one previous geostatistical study has used several Ab markers (namely PvAMA1, PvMSP1.19, PfAMA1 and Pf.GLURP.R2). However, in contrast with the current study, the researchers considered an individual positive when it responded for any of the two Ags for each species, not taking into account the differences in biological activity (e.g. longevity) among these Ags [20].

The previous study performed by Kerkhof et al. [26] has led to identification of serological markers with a relatively short half-life that were most likely to be reflective for recent exposure, such as *P. falciparum* Ags Pf.GLURP.R2, Pf.MSP1.19 and CSP. These serological markers could map the transmission risks with more precision and accuracy, as they provide the ability of distinguishing recent from past exposure [26, 41]. The current study, presented here, explored whether or not the use of serological markers is comparable to the use of PCR prevalence (asymptomatic cases) and malaria incidence data (symptomatic cases) to investigate spatial patterns in malaria transmission.

In Ratanakiri, significant malaria pockets were observed for both *P. falciparum* and *P. vivax* Ags. The largest pockets were located around the most northerly site of the ‘Tonle San River’ for all Ags. In comparison with the PCR prevalence data, *P. falciparum* exhibited similar pockets, whereas for *P. vivax* differences were seen. The similar pockets found between the PCR prevalence rates and sero-reactivity are in line with a study performed by Bousema et al. [16], that observed tight correlations as well.

When comparing the serologically based pockets with the incidence based pockets, the pockets neighbouring Vietnam were comparable, while the most northerly pockets at the ‘Tonle San River’ were slightly shifted to the West. There are malaria incidence based pockets found for *P. falciparum* situated around the capital ‘Ban Lung’ of the Ratanakiri province. Different studies [24, 42, 43] investigated the movement of individuals between villages, districts and countries. This might explain the malaria pockets seen around ‘Ban Lung’ raising the possibility that these individuals travel occasionally towards communities nearby the river or to remote areas. Overall, overlap was seen in the serological based pockets compared to the malaria incidence and parasite prevalence data.

That most pockets were perceived around the river confirms findings from other studies that also found more malaria pockets along open water bodies [19, 20, 44, 45]. The same pattern was observed by Sluydts et al. [25] who suggests that this is perhaps associated with increased movements of infected individuals and mosquito populations along the ‘Tonle San River’, and with the more remote location of these villages [25].

The specificity of serological markers for *P. falciparum* and *P. vivax* was acceptable (between 72 and 95 %) to predict PCR and malaria incidence pockets. However, sensitivity was in general much lower, except in predicting *P. falciparum* PCR pockets (between 74 and 95 %). In comparison, sensitivity of *P. falciparum* and *P. vivax* incidence in predicting PCR pockets lies between 10 and 50 %.

When looking at the different serological markers, variable patterns were observed, going from malaria pockets that move between the east and west in November 2012 and 2013 (CSP, Pf.MSP1.19 and Pv.MSP1.19) to lasting pockets that became smaller (Pf.GLURP.R2) or remained similar in size (PvAMA1). These varying patterns require further investigation related to the differences in immunogenicity and persistence of the Ab-responses [11, 46]. The only Ag that follows an expected altering pattern over time was Pf.GLURP.R2, which seems to correlate best with the PCR-prevalence and malaria incidence data. The latter is probably explained by the fact that this is a blood stage Ag with a short estimated Ab half-life [26]. This might reflect recent exposure with observing pockets that decline over time, suggesting that this serological marker might have potential in evaluating targeted malaria control efforts.

Risk factors related to sero-reactivity were identified by univariate and multivariable analyses. Significant elevated risks for *P. falciparum* malaria were seen for age, ethnicity and overnight stay at the plot hut. There were also differences observed in gender and sleeping in the forest, however, this is most probably negligible, as the IRR was very close to 1. Significant elevated risks for vivax markers were seen for age, whereas staying in plot huts showed to be a risk factor for Pv.MSP1.19 only. The *P. falciparum* outcomes are in line with a previous study performed in the same area by Sluydts et al. [25]. In this PCR prevalence based study that was performed on the baseline survey during the dry season, the most important risk factor detected was the overnight stay in the plot hut, based on both univariate and multivariable analyses. However, in the current serologically based study, it seems that age, concerning the older age groups, was the most important factor determining Ab-levels, compatible with cumulative exposure [7, 17, 26]. When immunity is acquired these Abs can persist for several years. This is caused by the presence of long-lived plasma cells that with every new exposure rapidly produce Abs against these parasites [47]. Therefore, when defining current exposure it is important to observe the Ab-levels in especially the younger age groups [17, 47]. Differences between *P. falciparum* and *P. vivax* could be explained by that fact that *P. vivax* shows relapse patterns that influence the serological outcomes, and that longer half-lives were observed for the *P. vivax* Ags in a previous study [20].

Overall, these outcomes confirm the findings of Sluydts et al. [25], and are also in line with the findings of Incardona et al. [48]. These researchers mentioned that entire families go together to the field and sleep in plot huts resulting in an increased exposure risk [25, 48]. Although this is not related to the age differences, as the age composition was similar inside and outside the pockets. However, this can be explained by the immunological maturity-status where children that acquire a malaria infection have the ability to boost their IgG titers, followed by a rapid decay [49]. These outcomes explain the population characteristics in the Greater Mekong Subregion, where ethnic minority groups, forest workers (of all ages) and migrants are known as the most widely recognized groups at risk [25, 50].

This study contributes in the validation of serological markers to distinguish very recent from past exposure, as suggested by Sturrock et al. [41]. This is especially the case for Pf.GLURP.R2, but also for Pf.MSP1.19, CSP and PvAMA1. By this means, combining more different Ags, covering the entire *Plasmodium* life cycle and having a longevity ranging from very short (~1 months) to long (year round), might lead to other promising results. However, methods to acquire the exact Ab-persistence are still in its infancy [20]. While PvAMA1 showed stable malaria pockets, Pf.GLURP.R2 suggest a decline in the remaining malaria pockets. The stability of pockets was also observed by Mosha et al. [11] on *P. falciparum* Ag AMA1 in the high endemic setting of Tanzania. Further development in quantifying exposure over different timescales, as well as the measurement of very recent exposure, serological approaches will provide a major contribution in estimating spatio-temporal patterns of risk [41]. The use of serology could benefit future malaria control programmes, since the use of serological markers can more precisely identify variation in transmission in low endemic areas. It should be noted that more serological markers that are competent to estimate exposure over different time-scales are required, as at present Pf.GLURP.R2 is most informative [41], as well as Pf.MSP1.19, CSP and PvAMA1 to a lesser extent. However, Pv.MSP1.19 should certainly not be ruled out, as it probably reflects transmission in the former past for which no PCR-prevalence data may be available.

## Conclusion

Identification of pockets with higher malaria transmission would be essential when adopting a malaria elimination strategy. Present study shows that PCR parasite prevalence, malaria incidence rates or serology, show equivalent results in identifying malaria pockets. The attempt to validate serological markers that are most likely to reflect current and past exposure led to Pf.GLURP.R2 showing a shrinking malaria pocket over time. Moreover, Pf.MSP1.19, CSP and PvAMA1 are also reflective for recent malaria transmission to a lesser extent. This means that serology can provide promising information for future research, especially in evaluating short-term interventions for malaria elimination. However, there is still more research required in selecting a promising combination of Ags, in particular for *P. vivax*.

## Additional files

**Additional file 1: Table S1.** Sensitivity and specificity analysis for the identification of malaria pockets.

**Additional file 2: Table S2.** Univariate analysis of risk factors per antigen and per Plasmodium species in Ratanakiri, Cambodia (2012 and 2013).
