# Supplementary figures and images for: Geographical patterns of malaria transmission based on serological markers for falciparum and vivax malaria in Ratanakiri, Cambodia
Source: Malar J. 2016 Oct 19;15:510. doi: 10.1186/s12936-016-1558-1 (PMC5069850; doi:10.1186/s12936-016-1558-1)

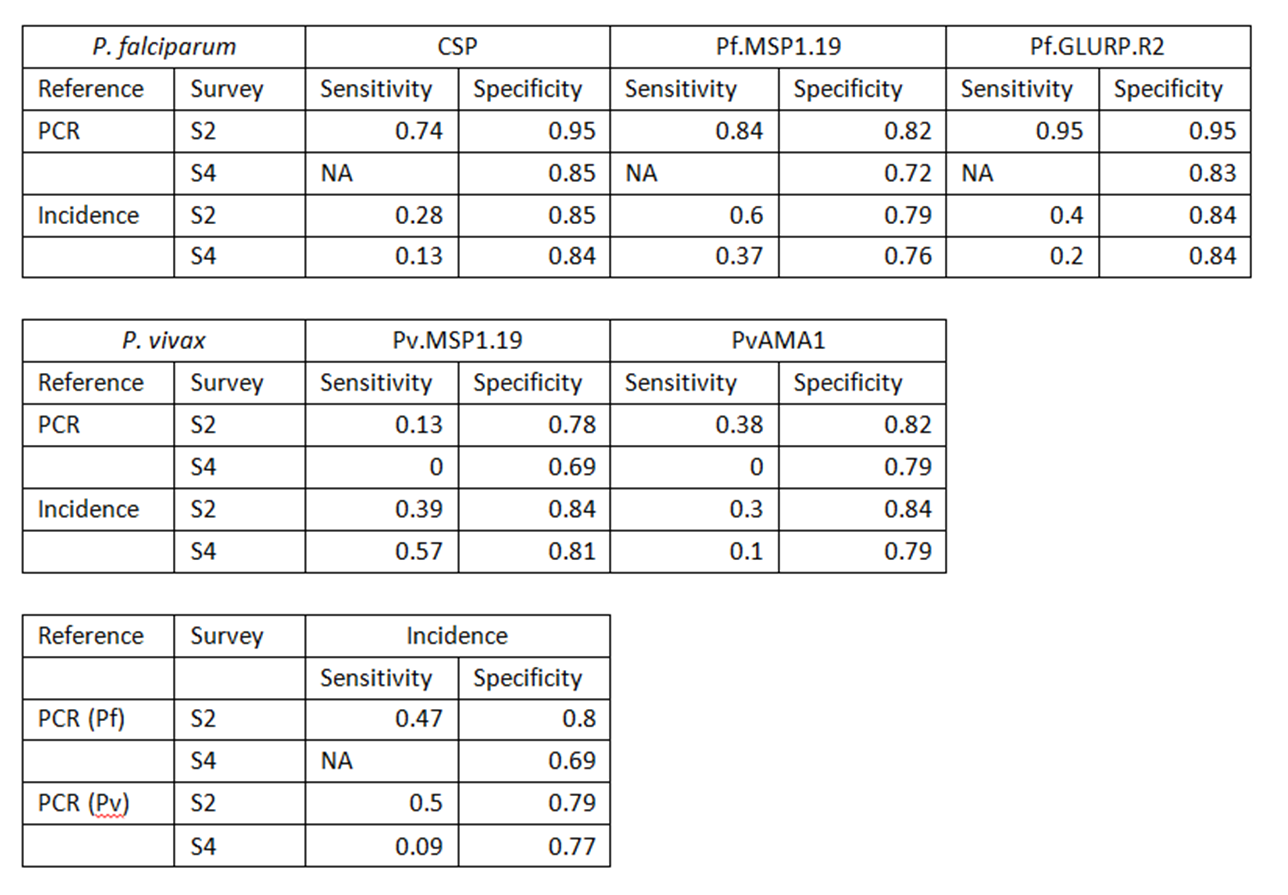

Supplement: Supplementary file 1 — Additional file 1: Table S1. Sensitivity and specificity analysis for the identification of malaria pockets. [file 12936_2016_1558_MOESM1_ESM.png]
